# Retraction: AI-assisted design of lightweight and strong 3D-printed wheels for electric vehicles

**DOI:** 10.1371/journal.pone.0327063

**Published:** 2025-06-25

**Authors:** The PLOS One Editors

After this article [1] was published, concerns were raised regarding the similarity to previous work [2] by different authors and that was not cited in [1].

Specifically, the overall concept, objectives, proposed approach, study design, and methodology appear highly similar to work reported in a published article [2]. The Methodology and Results and Discussion sections, including multiple figures reported in [1], appear very similar to content previously reported in [2], including several identical numerical values, though some text and figures are not identical.

In response to these concerns, the corresponding author stated that [2] was used as a basis for [1].

In light of the concerns about similarities between this article [1] and the previous work [2], the *PLOS One* Editors retract this article.

TOA, OOA, SAA, and AOO agreed with the retraction. AR and MOA either did not respond directly or could not be reached.

The retracted article [1] was removed from the *PLOS One* website at the time of retraction due to the similarities to [2]. The article’s Copyright and Data Availability statements were also updated at the time of retraction, and the removed contents are no longer offered under the Creative Commons Attribution License.
